# Synthesis and Bioactivity Evaluation of New 6-Aryl-5-cyano Thiouracils as Potential Antimicrobial and Anticancer Agents

**DOI:** 10.3390/molecules17089868

**Published:** 2012-08-17

**Authors:** Azza Taher Taher, Sahar Mahmoud Abou-Seri

**Affiliations:** 1 Department of Pharmaceutical Organic Chemistry, Faculty of Pharmacy, Cairo University, Kasr El-Aini Street, Cairo, P.O. Box, 11562, Egypt; Email: azzataher2005@yahoo.com; 2 Department of Pharmaceutical Chemistry, Faculty of Pharmacy, Cairo University, Kasr El-Aini Street, Cairo, P.O. Box, 11562, Egypt

**Keywords:** 6-aryl-5-cyano thiouracils, antibacterial, antifungal, anticancer

## Abstract

Several novel 6-aryl-5-cyano thiouracil derivatives were synthesized and explored for their activities as antibacterial, antifungal and anticancer agents. The antimicrobial evaluation revealed that compounds **7b** and **7c** possessed superior antibacterial activity against the Gram positive bacteria *S. aureus* and *B. subtilis* compared to the reference drug amoxicillin. Moreover, compound **4i** was found to be a broad spectrum antimicrobial agent and it also exhibited the highest antifungal activity against *C. albicans*, even higher than the reference drug amphotericin B (MIC = 2.34, 3.00 μg/mL respectively). Selected compounds were tested for *in vitro* cytotoxicity at a single 10^−5^ M concentration in accordance to the NCI (USA) protocol. The preliminary screening results showed that most of the compounds had limited cytotoxic activity against renal cancer UO-31 and/or A498 cell lines. Nevertheless, compounds **6d** and **6i** displayed potent growth inhibitory effect toward non-small cell lung cancer HOP-92 and leukemia MOLT-4 cell lines, respectively.

## 1. Introduction

**Figure 1:**
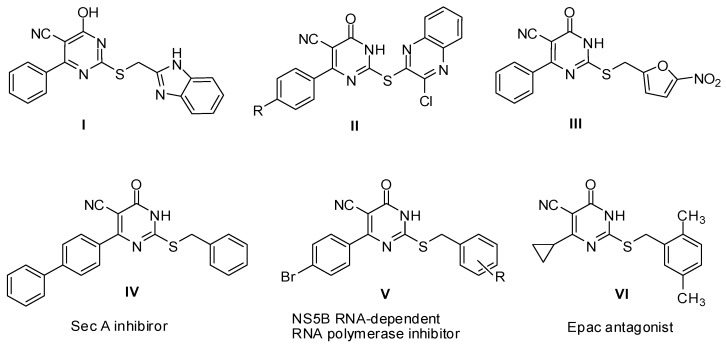
Structures of some potent antimicrobial and anticancer 6-aryl-5-cyano-2-thiouracil lead compounds.

Pyrimidines are an important component of nucleic acids and they have been used as building blocks in pharmaceuticals for the synthesis of antiviral [1], antineoplastic [2] antibacterial and antifungal [3] agents. Similarly, the related thiouracil derivatives are potential therapeutics as antiviral, anticancer and antimicrobial agents [4,5,6]. For example, *S*-alkylation and *N*-alkylation products have been recently reported as novel antibacterial, cytotoxic agents [7,8] and unique HIV reverse transcriptase inhibitors [9,10]. Moreover, a literature survey revealed that the thiouracilcarbonitrile ring system has occupied a marked position in the design and synthesis of novel chemotherapeutic agents with remarkable antitumor and antimicrobial activities (Figure 1). In particular, 2-[(1*H*-benzoimidazol-2-yl)methylthio]-4-hydroxy-6-phenylpyrimidine-5-carbonitrile (**I**) possessed significant broad spectrum antiproliferative activity *in vitro* [11]. In addition, thiouracil quinoxaline hybrids **II** demonstrated strong inhibitory effects on the EBV-EA activation with chemopreventive effect against carcinogenesis on Raji cells [12]. On the other hand, the nitrofuran analog **III** displayed a distinctive inhibitory activity against a panel of Gram positive bacteria [13]. Meanwhile, an array of 4-anilino- and 4-hydrazinothiopyrimidine-5-carbonitriles and their condensed heterocycles exerted promising chemotherapeutic activity as antimicrobial and anticancer agents [14,15,16]. Also, it was of great interest that specifically functionalized *S*-aralkylated 6-aryl-5-cyano-2-thiouracils may possess specific biological properties, including inhibition of bacterial protein translocase SecA (compound **IV**) [17], hepatitis C viral NS5B RNA dependent RNA polymerase (compound **V**) [18] and potent antagonist of Epac protein—a therapeutic target of cancer—(compound **VI**) [19].

In view of the biological significance of the above mentioned thiouracils, we herein report the synthesis and biological evaluation of novel 6-aryl-5-cyano-2-thiouracil derivatives **6a**–**i** and **7a**–**c** as potential antimicrobial and cytotoxic agents. The design of the target compounds **6a**–**i** was based on previous report that, for a series of 5-substituted-2-anilinopyrimidinones, a systematic increase in antimicrobial potency was observed upon elongation of the alkyl spacer between the phenyl ring and pyrimidinone pharmacophore from one to three carbons [20]. In analogy to this strategy, a new series of thiouracil-5-carbonitile derivatives **6a**–**i** was prepared in which structure modification was focused on changing the aryl methyl moiety in lead compounds **I**, **II** and **IV** to a bromobenzoylmethyl moiety and simultaneously introducing various substituted aryl groups at the 6 position of the thiouracil ring. The substituent on the aryl group was selected so as to confer different lipophilic and electronic environments on the molecules.

Furthermore, utilizing the active methylene site in **6** for incorporation of additional pharmacophoric group, the 4-hydroxyphenylhydrazono derivatives **7a**–**c** were prepared. Hydrazones represents an important class of compounds that show, besides broad spectrum antitumor activity, distinguished antifungal and antibacterial effects [21,22].

## 2. Results and Discussion

### 2.1. Chemistry

**Scheme 1:**
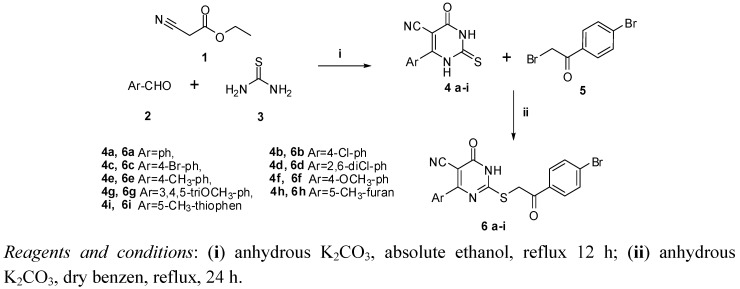
Synthetic pathway for target compounds **4a**–**i** and **6a**–**i**.

**Scheme 2:**
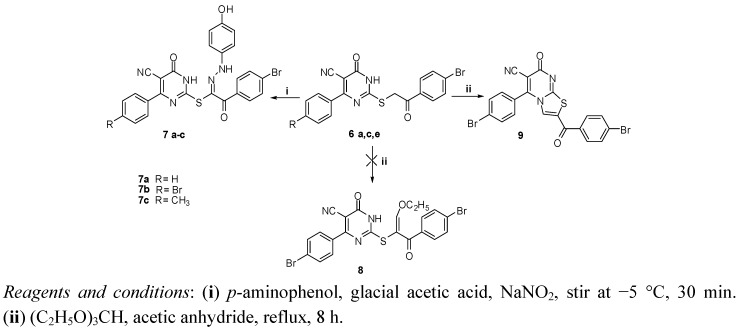
Synthetic pathways for compounds **7a**–**c** and **9**.

The synthetic approaches adopted to obtain the target compounds **6**–**9** are depicted in Schemes 1 and 2. The structures of the newly synthesized compounds were established on the basis of their elemental analyses and spectral data.

Ternary condensation of ethyl cyanoacetate **1** with the appropriate aldehyde **2** and thiourea **3** in the presence of anhydrous potassium carbonate afforded the 6-aryl-4-oxo-2-thioxo-1,2,3,4-tetrahydro-pyrimidine-5-carbonitrile(thiouracils) **4a**–**i**. The IR spectra of these compounds were characterized by the presence of NH stretching bands at 3410–3124 cm^−1^, C≡N bands at 2214–2152 cm^−1^ along with C=O bands at 1652–1625 cm^−1^ and C=S bands at 1253–1222 cm^−1^.

**Figure 2:**
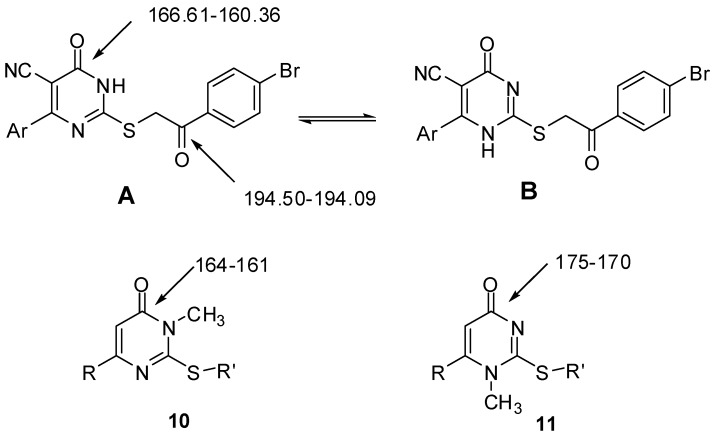
Possible tautomeric structures of compounds **6a**–**i** and ^13^C-NMR chemical shifts of reported thiouracils.

Selective *S*-alkylation of **4a**–**i** with bromophenacyl bromide **5** to produce compounds **6a**–**i** was carried out in refluxing dry benzene utilizing potassium carbonate as base catalyst (Scheme 1). The IR spectral data of compounds **6a**–**i** displayed no absorption bands for C=S, while an additional benzoyl C=O band was observed at 1735–1693 cm^−1^. Their ^1^H-NMR spectra revealed a singlet signal resonating at 5.98–4.51 ppm assignable to SCH_2_. Compounds **6a**–**i** may exist in one of two tautomeric forms **A** and **B** (Figure 2). To distinguish between these forms, ^13^C-NMR of compounds **6b**–**f** were recorded. The spectra showed two carbonyl signals corresponding to the pyrimidinone C=O at 166.61–160.36 and the benzoyl C=O at 194.50–194.09 ppm. Based on literature reports [23,24,25,26,27], the chemical shift of the pyrimidinone carbonyl is markedly affected by the nature of the adjacent nitrogen. The δ values of the pyrimidinone C=O in compounds **6b**–**f** suggest that N-(3) near to C=O is sp^3^-hybridized (pyrrole type) as it is similar to that found in the methyl derivative **10** and different from the C=O adjacent to sp^2^-hybridized nitrogen (pyridine type), which appears at 175–170 ppm (compound **11**) [26,27] (Figure 2). Accordingly, compounds **6a**–**i** are found as one tautomeric form namely, **A** rather **B**.

**Figure 3:**
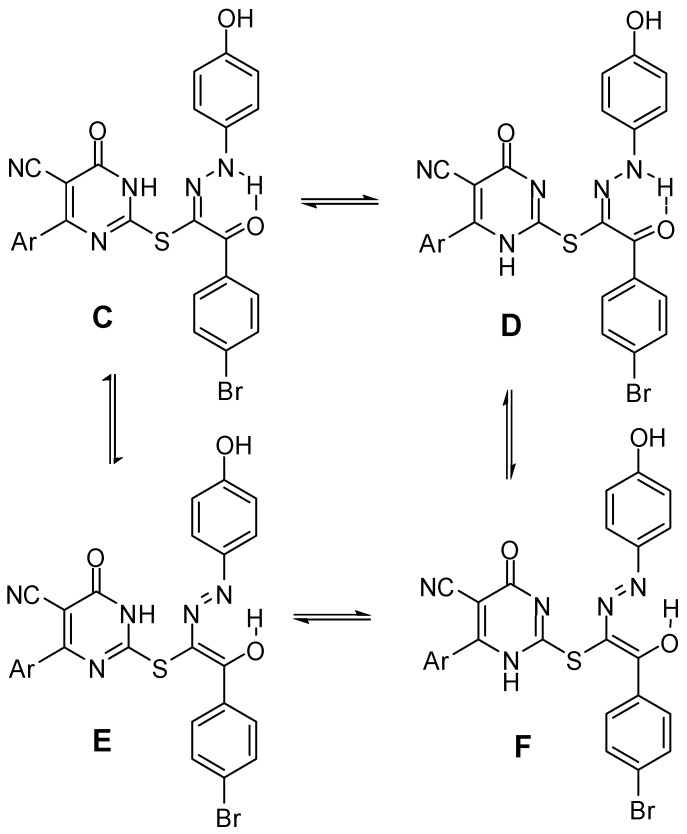
Possible tautomeric structures of compounds **7a**–**c**.

Reaction of compound **6a**, **6c** or **6e** with diazotized *p*-aminophenol in aqueous sodium hydroxide solution at −5 °C produced the corresponding arylhydrazono derivatives **7a**–**c** (Scheme 2). Compounds **7a**–**c** can exist in one or more of four tautomeric structures **C**–**F** (Figure 3). Their IR spectral data seem to be consistent more with the hydrazone structures (**C** or **D**) rather than the enolazo tautomeric forms (**E** or **F**). For example, all compounds exhibited two carbonyl bands in the regions 1654–1632 and 1670–1660 cm^−1^ corresponding to the stretching vibrations of the pyrimidinone and the benzoyl carbonyl groups, respectively. The low value of the wave number assigned for the latter C=O stretching band appears to result from chelation with NH and conjugation with the C=N double bond as required by hydrazone form **C** or **D** [28,29]. The ^1^H-NMR showed three exchangeable singlet signals at the range of 9.41–13.20 ppm due to the phenolic OH, the hydrazone NH and the pyrimidinone NH, while those derived from SCH_2_ were not detected. Finally, to differentiate between the **C** and **D** tautomers (Figure 3), the ^13^C-NMR spectrum of **7c** was recorded and compared with those of **6b**–**f**. The pyrimidinone C=O was detected at 161.04 ppm similar to those of **6b**–**f** and **10**. This finding indicates that the hydrazone derivatives **7a**–**c** exist predominantly in form **C**.

Refluxing **6c** with triethyl orthoformate in acetic anhydride gave the thiazolo[3,2-a]pyrimidine derivative **9** instead of the expected ethoxy methene derivative **8**, as a result of intramolecular cyclization of the latter (Scheme 2). The ^1^H-NMR spectrum of the product revealed the disappearance of SCH_2_ signal and the appearance of a singlet signal at 7.65 ppm assigned for the thiazole CH. A notable feature in the ^1^H-NMR spectrum was the absence of the triplet-quartet pattern of the ethoxy group and D_2_O exchangeable (NH) signal, which asserted the production of the cyclic thiazolo[3,2-a]pyrimidine derivative **9**.

### 2.2. Biological Evaluation

#### 2.2.1. Antimicrobial Activity

**Figure 4:**
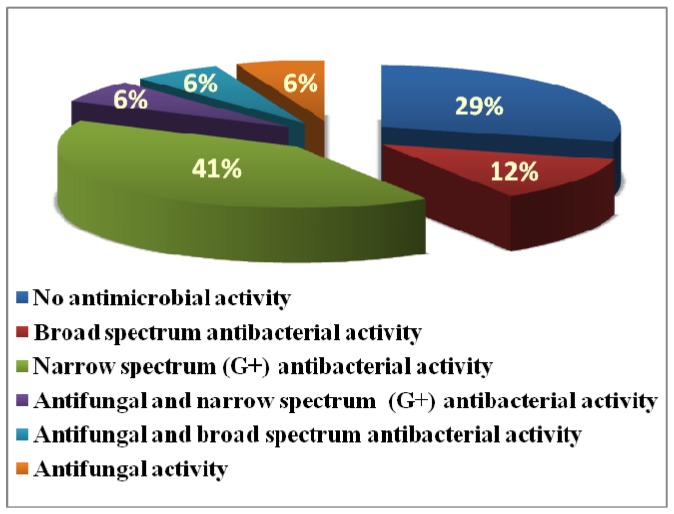
The antimicrobial spectrum of tested compounds.

The newly synthesized compounds were evaluated for their *in vitro *antibacterial activity against *Staphylococcus aureus* ATCC 6538P, *Bacillus subtilis* ATCC CC33, *Escherichia coli *ATCC 5087 and *Pseudomonas aeruginosa *ATCC 9027, as well as for their antifungal activity against *Candida albicans* ATCC 60193 *and Aspergillus niger *ATCC 1718109 using the microbroth dilution method [30]. The minimum inhibitory concentration (MIC) and concentration that inhibit 50% of microorganisms (IC_50_) as measures of the microbial inhibitory activity as well as minimum bactericidal concentration (MBC) that reflects the bactericidal activity of the tested compounds were calculated at μg/mL (Table1). The data presented in Table 1 revealed that compounds **6a** and **6c** had good to fair broad spectrum antibacterial activity, other compounds **4d**, **4h**, **6f**, **6g**, **7a**–**c** were only active against Gram positive strains. As for antifungal activity, compound **4g** exhibited moderate activity against *C. albicans* and *A. niger*, while **9** elicited weak antifungal activity against *A. niger *with Gram positive antibacterial activity. Among the tested compounds, only compound **4i** displayed pronounced broad spectrum antibacterial and antifungal activities. On the other hand, the remaining compounds **6b**, **6d**, **6e**, **6h** and **6i** had no significant activity against any of the tested strains at concentration up to 50 μg/mL (Figure 4).

**Table 1:** Antimicrobial activity of the synthesized compounds expressed as minimum inhibitory concentration (MIC), minimum bactericidal concentration (MBC) and concentration that inhibit 50% of microorganisms (IC_50_) in µg /mL against the pathological strains based on two fold serial dilution technique.

Compound		Gram positive bacteria		Gram negative bacteria		Fungi	
		*S. aureus*	*B. subtlis*	*E. coli*	*P. aeruginosa*	*C. albicans*	*A. niger*
**4d**	MIC	**9.38**	**9.38**	>50	>50	>50	>50
	MBC	**9.30**	**9.30**	>50	>50	>50	>50
	IC_50_	**4.20**	**6.25**	>50	>50	>50	>50
**4g**	MIC	>50	>50	>50	>50	**9.38**	**18.75**
	MBC	>50	>50	>50	>50	**9.30**	**18.75**
	IC_50_	>50	>50	>50	>50	**6.25**	**12.50**
**4h**	MIC	**1.17**	**1.17**	>50	>50	>50	>50
	MBC	**1.56**	**1.56**	>50	>50	>50	>50
	IC_50_	**0.40**	**0.78**	>50	>50	>50	>50
**4i**	MIC	**2.34**	**9.38**	**9.38**	**9.38**	**2.34**	**4.69**
	MBC	**2.30**	**9.30**	**9.30**	**9.30**	**2.30**	**6.25**
	IC_50_	**1.17**	**6.25**	**3.13**	**3.13**	**1.17**	**3.13**
**6a**	MIC	**4.69**	**9.38**	**18.75**	**37.50**	>50	>50
	MBC	**3.80**	**9.00**	**18.75**	**37.50**	>50	>50
	IC_50_	**2.30**	**6.25**	**12.50**	**25.00**	>50	>50
**6b**	MIC	>50	>50	>50	>50	>50	>50
	MBC	>50	>50	>50	>50	>50	>50
	IC_50_	>50	>50	>50	>50	>50	>50
**6c**	MIC	**37.50**	**37.50**	**37.50**	**37.50**	> 50	> 50
	MBC	**37.50**	**37.50**	**37.50**	**37.50**	> 50	> 50
	IC_50_	**12.50**	**25.00**	**25.00**	**25.00**	> 50	> 50
**6d**	MIC	>50	>50	>50	>50	>50	>50
	MBC	>50	>50	>50	>50	>50	>50
	IC_50_	>50	>50	>50	>50	>50	>50
**6e**	MIC	>50	>50	>50	>50	>50	>50
	MBC	>50	>50	>50	>50	>50	>50
	IC_50_	>50	>50	>50	>50	>50	>50
**6f**	MIC	**2.34**	**4.69**	>50	>50	>50	>50
	MBC	**3.13**	**6.25**	>50	>50	>50	>50
	IC_50_	**1.56**	**3.13**	>50	>50	>50	>50
**6g**	MIC	**4.69**	**4.69**	>50	>50	>50	>50
	MBC	**4.70**	**4.70**	>50	>50	>50	>50
	IC_50_	**1.56**	**1.56**	>50	>50	>50	>50
**6h**	MIC	>50	>50	>50	>50	>50	>50
	MBC	>50	>50	>50	>50	>50	>50
	IC_50_	>50	>50	>50	>50	>50	>50
**6i**	MIC	>50	>50	>50	>50	>50	>50
	MBC	>50	>50	>50	>50	>50	>50
	IC_50_	>50	>50	>50	>50	>50	>50
**7a**	MIC	**18.75**	**18.75**	>50	>50	>50	>50
	MBC	**18.75**	**18.75**	>50	>50	>50	>50
	IC_50_	**12.50**	**12.50**	>50	>50	>50	>50
**7b**	MIC	**1.17**	**2.34**	>50	>50	>50	>50
	MBC	**1.17**	**2.30**	>50	>50	>50	>50
	IC_50_	**0.78**	**1.17**	>50	>50	>50	>50
**7c**	MIC	**0.19**	**1.17**	>50	>50	>50	>50
	MBC	**0.20**	**1.17**	>50	>50	>50	>50
	IC_50_	**0.15**	**0.40**	>50	>50	>50	>50
**9**	MIC	**37.50**	**37.50**	>50	>50	>50	**37.50**
	MBC	**37.50**	**50.00**	>50	>50	>50	**50.00**
	IC_50_	**12.50**	**37.50**	>50	>50	>50	**37.50**
**Amoxicillin**	MIC	1.25	150.00	NA	NA	NA	NA
**Gentamicin**	MIC	NA	NA	1.00	8.00	NA	NA
**Amphotericin B**	MIC	NA	NA	NA	NA	3.00	1.25

The structure activity correlation of the tested compounds showed that, the starting 6-aryl-5-cyano-2-thiouracil derivatives **4d**, **4g**–**i** had moderate to potent antimicrobial activity. Both the antimicrobial spectrum and potency of such compounds seem to be dependent on the the aryl substituent at the 6 position of the thiouracil ring. Compound **4d** with a 2,6-dichlorophenyl was moderately active against Gram positive bacteria only. An eight-fold increase in inhibitory activity against the same bacterial isolates was observed with methylfuran analog **4h**. Appending a methylthiophene to the thiouracil ring as in **4i** changed the antimicrobial spectrum from narrow to broad. *S. aureus *was the most sensitive bacteria to **4i** followed by *B. subtilis*, *E. coli *and *P. aeruginosa*. Moreover, it exhibited the highest antifungal activity against *C. albicans*, even stronger than the reference drug amphotericin B (MIC = 2.34, 3.00 μg/mL respectively) and fairly potent activity against *A. niger*. On the other hand, modest antifungal activity was observed for the trimethoxyphenyl congener **4g**.

Considering the *S*-bromobenzoylmethyl thiouracil derivatives **6a**–**i**, they proved to have no antifungal activity. This suggested that *S*-alkylation has a negative impact on the antifungal activity of the synthesized thiouracils (**4g** *versus* **6g** and **4i** *versus* **6i**). On the other hand, the antibacterial activity of such compounds appears to be affected by the nature of aryl substituent on the thiouracil scaffold. Compound **6a** with an unsubstituted phenyl group displayed good activity against *S. aureus* and *B. subtilis*, in addition to moderate to low activity against the Gram-negative *E. coli *and *P. aeruginosa*. Meanwhile, the presence of a phenyl-bearing mesomeric electron-donating methoxy group resulted in compounds **6f** and **6g** with enhanced activity against Gram positive bacteria only. Conversely, analogs with heteroaryl substituent **6h** and **6i** or phenyl attached to inductive electron-donating or electron withdrawing atoms as in **6b**–**e** were either inactive or weakly active against the investigated bacteria, assuming that the electronic nature of the substituents is of major importance.

The incorporation of a 4-hydroxyphenylhydrazono moiety to the active methylene in **6a**, **6c** and **6e** produced **7a**–**c** with remarkable inhibition effect against Gram positive *S. aureus* and *B. subtilis. *Compound **7a** with phenyl substituent at the 6-postion of the thiouracil ring possessed moderate activity. However, the more lipophilic *p*-bromophenyl **7b** and *p*-tolyl **7c** analogs had superior antibacterial activity compared to the reference drug amoxicillin. In particular, **7c** being the most potent antibacterial agent in this study with MIC 0.19 and 1.17 μg/mL against *S. aureus* and *B. subtilis*, respectively, indicating that, the presence of an electron-donating methyl group is more favorable for the antibacterial effect of this class of compounds than electron-withdrawing bromine atom. Also, the excellent antibacterial activity of **7a**–**c** might be attributed to the presence of 4-hydroxyphenylhydrazono moiety. It has been reported [31,32,33], that phenolic compounds can sensitize the phospholipid bilayer of the microbial cytoplasmic membrane causing increased permeability, unavailability of vital intracellular constituents and/or impairment of bacterial enzyme systems related to energy production.

The cyclization of **6c** into the corresponding thiazolo[3,2-a]pyrimidine derivative **9** affected the microbial spectrum rather than potency. While, **6c** proved to have mild broad spectrum antibacterial activity with no antifungal activity, **9** demonstrated weak antimicrobial activities toward Gram positive bacterial strains and the fungus *A. niger*. Finally, the narrow concentration difference between the MIC and MBC of active compounds indicated that they have bactericidal effects rather than bacteriostatic ones.

#### 2.2.2. Anticancer Activity

Thirteen compounds **4d**, **4g**–**i** and **6a**–**i** were selected by the National Cancer Institute (NCI) Developmental Therapeutic Program (www.dtp.nci.nih.gov) to be screened for their anticancer activity *in vitro. *The anticancer assays were performed in accordance with the protocol of the Drug Evaluation Branch, NCI, Bethesda [34,35,36]. The compounds were first evaluated at one dose primary anticancer assay towards a panel of approximately 60 cancer lines (concentration 10^−5^ M). The human tumor cell lines were derived from nine different cancer types: leukemia, melanoma, lung, colon, central nervous system (CNS), ovarian, renal, prostate and breast cancers. A 48 h drug exposure protocol was used and sulforhodamine B (SRB) protein assay was applied to estimate the cell viability and growth [37]. Results for each tested agent were reported as the percentage growth of the treated cells compared to the untreated control cells and also, presented as mean graph of the growth present. The preliminary screening results (Table 2) showed that most of the compounds had limited cytotoxic activity against the tested cell lines with percentage growth ranging from 73.09 to 133.71%. Nevertheless, compounds **6d** and **6i** displayed potent growth inhibitory potency toward non-small cell lung cancer HOP-92 and leukemia MOLT-4 with growth % 41.03 and 42.38, respectively. 

Regarding sensitivity of individual cell lines, all the evaluated compounds exhibited a distinguished sensitivity profile toward one or more of the renal cancer cell lines in comparison with other cell lines, namely UO-31, A498 or 786-0. Moreover, compounds **4i** and **6i** restrained the growth of melanoma LOX IMVI cancer cell line. It is noteworthy that both compounds have highly lipophilic methylthiophen motif as substituent on thiouracil moiety, which might increase their availability and penetration to melanoma cells. In addition, compounds **6d** and **6i** were found especially effective against non-small cell lung cancer HOP-92 and leukemia MOLT-4, respectively. 

Structurally, elaborating the thiouracils **4g**–**i** with bromobenzoylmethyl moiety in **6g**–**i** led to compounds that had comparable or slightly better inhibitory efficacy with almost similar antitumor spectrum. For instance, the analogue **6i** exhibited a marginal activity increase toward the same melanoma and renal cancer cell lines as **4i**, in addition to remarkable high activity against MOLT-4 leukemia cell line (growth % 42.38). Interestingly, applying the same structure modification to **4d **afforded **6d** with a unique broad anticancer spectrum. The latter, elicited the highest growth inhibitory potential against seven tumor sub-panels; leukemia, colon cancer, CNS cancer, melanoma, renal cancer and breast cancer with noticeable antiproliferative effect on non small lung cancer HOP-92 (growth % 41.03).

Eventually, it seems that the position rather than electronic nature of substituents on 6-aryl group of thiouracil ring had a prominent effect on the activity profile of compounds **6a**–**i**. The steric hindrance generated by the *ortho-ortho* dichloro substituents on the 6-aryl in **6d** can create constrain and impose particular orientation of the aryl group relative to the rest of the molecule. Such conformation might be favorable to cytotoxicity and could explain the observed activity of **6d**. In brief, the coexistence of methylthiophene or 2,6-dichlophenyl at position 6 and bromobezoylmethyl moiety at position 2 of the thiouracil ring increases the cytotoxic activity against specific cell lines.

**Table 2:** Cytotoxic activity of the tested compounds against a panel of 60 cancer cell lines at 10 µM concentration.

Compound	Mean growth, %	Range of growth, %	Panel	Most sensitive cell lines
				growth, %
**4d**	102.9	76.85–126.64	Renal Cancer	UO-31 (76.85)
**4g**	102.53	81.19–121.32	Renal Cancer	A498 (81.19)
**4h**	104.95	82.37–133.71	Renal Cancer	UO-31 (82.37)
**4i**	105.24	76.99–127.08	Melanoma	L OX IMVI (76.99)
			Renal Cancer	786-0 (83.54) , UO-31 (84.31)
**6a**	102.14	80.39–119.48	Renal Cancer	A498 (85.49) , UO-31 (80.39)
**6b**	103.26	80.27–121.43	Renal Cancer	A498 (80.27)
**6c**	102.26	80.07–117.90	Renal Cancer	A498 (80.07)
**6d**	89.03	41.03–118.60	Leukemia	HL-60(TB) (75.34), K-562 (73.85), MOLT-4 (73.33),
				RPMI-8226 (72.35), SR (79.59).
				EKVX (79.94),** HOP-92 (41.03)**, NCI-H522 (75.25).
			Non Small Cell Lung Cancer	HCT-116 (74.54),
				HTC-15 (80.89).
				SF-295 (81.77).
			Colon Cancer	SK-MEL-2 (82.18),
			CNS Cancer	UACC-62 (80.12)
			Melanoma	780-0 (73.64), A498 (75.93), CAKI-1 (71.34), UO-31 (80.25).
			Renal Cancer	MCF-7 (83.81), T-47D (74.23), MDA-MB-468 (73.27)
	
			Breast Cancer	
**6e**	101.92	82.81–119.49	Renal Cancer	UO-31 (82.81)
**6f**	102.3	78.50–121.80	Non-Small Cell Lung Cancer	HOP-92 (85.86)
			Renal Cancer	
				A498 (78.50), UO-31 (86.29)
**6g**	102.98	83.81–124.78	Renal Cancer	A498 (84.19), UO-31 (83.81)
**6h**	101.72	73.09–114.65	Renal Cancer	UO-31 (73.09)
**6i**	102.41	42.38–119.33	Leukemia	**MOLT-4 (42.38)**
			Melanoma	L OX IMVI (75.06)
			Renal Cancer	786-0 (81.16), UO-31 (80.26)

## 3. Experimental

### 3.1. Chemistry

Melting points are uncorrected and determined in one end open capillary tubes using Gallenkamp melting point apparatus MFB-595-010M (Gallenkamp, London, England). Microanalysis was carried out at Micro-analytical Unit, Regional Centre for Microbiology and Biotechnology, Al-Azhar University. Infrared spectra were recorded on a Shimadzu FT-IR 8400S spectrophotometer (Shimadzu, Kyoto, Japan), using potassium bromide discs and results are expressed in wave numbers (cm^−1^). The NMR spectra were recorded on a Varian Mercury VX-300 NMR spectrometer. ^1^H- spectra were run at 300 MHz and ^13^C- spectra were run at 75.46 MHz in dimethylsulphoxide (DMSO-*d_6_*). Chemical shifts are quoted in δ and were related to that of the solvents. Mass spectra were recorded using a Hewlett Packard Varian (Varian, Palo Alto, CA, USA) and Shimadzu Gas Chromatograph Mass Spectrometer-QP 1000 EX (Shimadzu, Kyoto, Japan). TLC were carried out using Art.DC-Plastikfolien, Kieselgel 60 F254 sheets (Merck, Darmstadt, Germany), the developing solvents were chloroform/methanol (9:1) and the spots were visualized at 366 and 254 nm by UV Vilber Lourmat 77,202 (Vilber, Marne La Vallee, France). Compounds **4a**–**c** and **4e**–**g** were obtained according to the reported procedures [15,18,38,39,40], while compound **6a** is commercially available. 

*6-Aryl-4-oxo-2-thioxo-1,2,3,4-tetrahydropyrimidine-5-carbonitriles* **4a**–**i**. A mixture of ethyl cyanoacetate **1** (1.13 g, 1.07 mL, 0.01 mol), thiourea **3** (0.76 g, 0.01mol), anhydrous potassium carbonate (2.07 g, 0.015 mol) and the appropriate aromatic aldehyde **2** (0.01 mol) in absolute ethanol (25 mL) was heated under reflux for 12 h. The reaction mixture was allowed to cool and the formed precipitate was filtered. The residue was triturated with water and neutralized with acetic acid. The precipitate was filtered, washed twice with cold water (20 mL), dried, and crystallized from ethanol.

*6-(2,6-Dichlorophenyl)-4-oxo-2-thioxo-1,2,3,4-tetrahydropyrimidine-5-carbonitrile* (**4d**): Yield 55%, m.p: 261–263 °C. ^1^H-NMR: *δ*: 7.48–7.60 (3H, m), 7.63 (1H, d, *J *= 7.8), 7.86 (1H, d, *J *= 6.9). ^13^C-NMR *δ*:125.03, 128.18, 128.37, 129.09, 130.97, 137.35, 159.94, 160.00, 176.13. IR cm^−1^: 3344 and 3124 (NH), 3012 (CH aromatic), 2939 and 2900 (CH aliphatic), 2206 (C≡N), 1631 (C=O), 1253 (C=S). Anal. Calcd. for C_11_H_5_Cl_2_N_3_OS (298.15): C, 44.31; H, 1.69; N, 14.09. Found: C, 44.61; H, 1.77; N, 14.28.

*4-Oxo-2-thioxo-6-(3,4,5-trimethoxyphenyl)-1,2,3,4-tetrahydropyrimidine-5-carbonitrile* (**4g**) [15]: Yield 62%, m.p: 250–252 °C, (reported m.p: 245–247 °C). ^1^H-NMR *δ*: 3.73 (3H, s), 3.82 (6H, s), 6.61 (2H, s), 11.53 (2H, br s). ^13^C-NMR *δ*: 55.69, 60.05, 84.84, 106.27, 119.05, 132.64, 138.95, 152.13, 152.54, 162.54, 166.65, 182.56. IR cm^−1^: 3344 and 3294 (NH), 3136 (CH aromatic), 2954 and 2839 (CH aliphatic), 2152 (C≡N), 1627 (C=O), 1246 (C=S). Anal. Calcd. for C_14_H_13_N_3_O_4_S (319.34): C, 52.66; H, 4.10; N, 13.16. Found: C, 52.86; H, 4.23; N, 13.52.

*6-(5-Methylfuran-2-yl)-4-oxo-2-thioxo-1,2,3,4-tetrahydropyrimidine-5-carbonitrile* (**4h**): Yield 48%, m.p: 242 °C decomp. ^1^H-NMR *δ*: 2.36 (3H, s), 6.33 (1H, d, *J *= 2.2), 7.20 (1H, d, *J *= 2.2), 11.47 (2H, br s, D_2_O exchangeable). IR cm^−1^: 3394, br (NH), 3039 (CH aromatic), 2924 and 2854 (CH aliphatic), 2214 (C≡N), 1652 (C=O), 1222 (C=S). Anal. Calcd. for C_10_H_7_N_3_O_2_S (233.03): C, 51.49; H, 3.02; N, 18.02. Found: C, 51.62; H, 3.14; N, 18.09.

*6-(5-Methylthiophen-2-yl)-4-oxo-2-thioxo-1,2,3,4-tetrahydropyrimidine-5-carbonitrile* (**4i**): Yield 45%, m.p: 201–203 °C. ^1^H-NMR *δ*: 2.48 (3H, s), 6.89 (1H, d, *J *= 3.6), 7.44 (1H, d, *J *= 3.9), 11.45 (2H, br s, D_2_O exchangeable). IR cm^−1^: 3410, br (NH), 3093 (CH aromatic), 2920 and 2854 (CH aliphatic), 2210 (C≡N), 1625 (C=O), 1242 (C=S). Anal. Calcd. for C_10_H_7_N_3_OS_2_ (249.31): C, 48.18; H, 2.83; N, 16.85. Found: C, 48.60; H, 2.90; N, 17.31. MS *m/z*: 249 [M]^+^.

*6-Aryl-2-(4-bromobenzoylmethylthio)-4-oxo-3,4-dihydropyrimidine-5-carbonitriles* **6a**–**i**. To a solution of thiouracil derivatives **4a**–**i** (0.01 mol) in dry benzene (10 mL) was added anhydrous potassium carbonate (4.14 g, 0.03 mol) and bromophenacyl bromide **5** (2.78 g, 0.01 mol). The mixture was heated under reflux for 24 h. Then, the solvent was reduced under *vacuum* and the reaction mixture was cooled. The precipitate was filtered, washed twice with cold water, dried and crystallized from methanol.

*2-(4-Bromobenzoylmethylthio)-4-oxo-6-phenyl-3,4-dihydropyrimidine-5-carbonitrile* (**6a**): Yield 68%, m.p: 242–244 °C. IR cm^−1^: 3429, br (NH), 3086 (CH aromatic), 2920 and 2854 (CH aliphatic), 2194 (C≡N), 1701 and 1631 (C=Os). Anal. Calcd. for C_19_H_12_BrN_3_O_2_S (426.29): C, 53.53; H, 2.84; N, 9.86. Found: C, 53.42; H, 2.64; N, 10.14. MS *m/z*: 425 [M−1]^+^.

*2-(4-Bromobenzoylmethylthio)-6-(4-chlorophenyl)-4-oxo-3,4-dihydropyrimidine-5-carbonitrile* (**6b**):Yield 62%, m.p: 248–250 °C. ^1^H-NMR *δ*: 4.55 (2H, s), 7.40 (2H, d, *J *= 8.1), 7.63 (2H, d, *J *= 8.1), 7.71 (2H, d, *J *= 8.4), 7.94 (2H, d, *J *= 8.4), 11.75 (1H, br s, D_2_O exchangeable). ^13^C-NMR *δ*: 37.26, 89.06, 119.69, 127.21, 127.92, 129.73, 130.24, 131.60, 134.32, 135.25, 136.12, 165.49, 169.91, 170.69, 194.15. IR cm^−1^: 3371, br (NH), 3105 (CH aromatic), 2916 (CH aliphatic), 2194 (C≡N), 1697 and 1650 (C=Os). Anal. Calcd. for C_19_H_11_BrClN_3_O_2_S (460.73): C, 49.53; H, 2.41; N, 9.12. Found: C, 49.46; H, 2.61; N, 9.42. MS *m/z*: 459 [M]^+^, 461 [M+2]^+^, 463 [M+4]^+^.

*6-(4-Bromophenyl)-2-(4-bromobenzoylmethylthio)-4-oxo-3,4-dihydropyrimidine-5-carbonitrile* (**6c**):Yield 68%, m.p: 250–252 °C. ^1^H-NMR *δ*: 4.55 (2H, s), 7.52 (2H, d, *J *= 8.1), 7.59–7.82 (4H, m), 7.93 (2H, d, *J *= 8.4), 11.70 (1H, br s). ^13^C-NMR *δ*: 37.26, 88.97, 119.71, 123.085, 123.54, 127.23, 128.41, 129.77, 130.19, 130.85, 131.59, 135.20, 162.37, 169.86, 170.72, 194.09. IR cm^−1^: 3421, br (NH), 3050 (CH aromatic), 2916 (CH aliphatic), 2194 (C≡N), 1693-1628 (C=Os). Anal. Calcd. for C_19_H_11_Br_2_N_3_O_2_S (505.18): C, 45.17; H, 2.19; N, 8.32. Found: C, 45.19; H, 2.34; N, 8.47. MS *m/z*: 504 [M+1]^+^, 505 [M+2]^+^, 506 [M+3]^+^, 507 [M+4]^+^.

*2-(4-Bromobenzoylmethylthio)-6-(2,6-dichlorophenyl)-4-oxo-3,4-dihydropyrimidine-5-carbonitrile* (**6d**): Yield 65%, m.p: > 300 °C. ^1^H-NMR *δ*: 5.98 (2H, s), 7.23 (3H, m), 7.37 (4H, d, *J *= 7.8), 8.74 (1H, s, D_2_O exchangeable). ^13^C-NMR *δ*: 107.49, 116.46, 119.40, 123.17, 127.45, 128.39, 131.11, 132.54, 133.15, 135.26, 141.72, 147.03, 163.17, 169.64, 194.50. IR cm^−1^: 3197, br (NH), 3128 (CH aromatic), 2974 (CH aliphatic), 2160 (C≡N), 1735 and 1619 (C=Os). Anal. Calcd. for C_19_H_10_BrCl_2_N_3_O_2_S (495.18): C, 46.09; H, 2.04; N, 8.49. Found: C, 46.13; H, 2.33; N, 8.86. MS *m/z*: 493 [M]^+^, 494 [M+1]^+^, 495 [M+2]^+^, 496 [M+3]^+^.

*2-(4-Bromobenzoylmethylthio)-4-oxo-6-p-tolyl-3,4-dihydropyrimidine-5-carbonitrile* (**6e**): Yield 70%, m.p: 262–264 °C. ^1^H-NMR *δ*: 2.32 (3H, s), 4.89 (2H, s), 7.07 (2H, d, *J *= 8.1), 7.50 (2H, d, *J *= 8.1), 7.75 (2H, d, *J *= 8.4), 7.95 (2H, d, *J *= 8.4), 11.30 (1H, br s, D_2_O exchangeable). ^13^C-NMR *δ*: 20.85, 37.23, 88.78, 120.06, 127.19, 128.13, 128.43, 130.25, 131.59, 134.57, 135.25, 139.22, 166.61, 170.27, 170.43, 194.23. IR cm^−1^: 3387, br (NH), 3012 (CH aromatic), 2916 (CH aliphatic), 2194 (C≡N), 1701 and 1631 (C=Os). Anal. Calcd. for C_20_H_14_BrN_3_O_2_S (440.31): C, 54.56; H, 3.20; N, 9.54. Found: C, 54.83; H, 3.29; N, 9.82. MS *m/z*: 439 [M]^+^, 441 [M+2]^+^.

*2-(4-Bromobenzoylmethylthio)-6-(4-methoxyphenyl)-4-oxo-3,4-dihydropyrimidine-5-carbonitrile* (**6f**): Yield 75%, m.p: 285–287 °C. ^1^H-NMR *δ*: 3.82 (3H, s), 4.56 (2H, s), 6.87 (2H, d, *J *= 6.9), 7.63 (2H, d, *J *= 6.9), 7.72 (2H, d, *J *= 6.9), 7.95 (2H, d, *J *= 6.6), 11.55 (1H, br s). ^13^C-NMR *δ*: 37.27, 55.20, 88.25, 113.18, 120.35, 127.24, 129.56, 129.64, 129.66, 130.33, 131.66, 135.25, 160.36, 166.02, 170.36, 194.22. IR cm^−1^: 3394, br (NH), 3012 (CH aromatic), 2916 (CH aliphatic), 2194 (C≡N), 1697 and 1635 (C=Os). Anal. Calcd. for C_20_H_14_BrN_3_O_3_S (456.31): C, 52.64; H, 3.09; N, 9.21. Found: C, 52.28; H, 3.19; N, 9.53. MS *m/z*: 455 [M]^+^, 457 [M+2]^+^. 

*2-(4-Bromobenzoylmethylthio)-4-oxo-6-(3,4,5-trimethoxyphenyl)-3,4-dihydropyrimidine-5-carbonitrile* (**6g**): Yield 55%, m.p: > 300 °C. ^1^H-NMR *δ*: 3.80 (3H, s), 3.82 (3H, s), 3.84 (3H, s), 5.23 (2H, s), 7.06 (1H, s), 7.19 (1H, s), 7.39 (2H, d, *J *= 8.7), 7.85 (2H, d, *J *= 8.7), 10.10 (1H, br s, D_2_O exchangeable). IR cm^−1^: 3425, br (NH), 3012 (CH aromatic), 2924 (CH aliphatic), 2200 (C≡N), 1701 and 1635 (C=Os). Anal. Calcd. for C_22_H_18_BrN_3_O_5_S (516.36): C, 51.17; H, 3.51; N, 8.14. Found: C, 51.29; H, 3.22; N, 8.54. MS *m/z*: 515 [M]^+^.

*2-(4-Bromobenzoylmethylthio)-6-(5-methylfuran-2-yl)-4-oxo-3,4-dihydropyrimidine-5-carbonitrile* (**6h**): Yield 40%, m.p: 192 °C decomp. ^1^H-NMR *δ*: 2.33 (3H, s), 4.55 (2H, s), 7.15 (1H, d, *J *= 3.6), 7.42 (2H, d, *J *= 8.1), 7.54 (1H, d, *J *= 3.6), 7.77 (2H, d, *J *= 8.1), 11.50 (1H, br s, D_2_O exchangeable). IR cm^−1^: 3441, br (NH), 3089 (CH aromatic), 2912 (CH aliphatic), 2218 (C≡N), 1693 and 1658 (C=Os). Anal. Calcd. for C_18_H_12_BrN_3_O_3_S (430.28): C, 50.25; H, 2.81; N, 9.77. Found: C, 50.18; H, 2.76; N, 9.97. MS *m/z*: 429 [M]^+^, 431 [M+2]^+^.

*2-(4-Bromobenzoylmethylthio)-6-(5-methylthiophen-2-yl)-4-oxo-3,4-dihydropyrimidine-5-carbonitrile* (**6i**): Yield 45%, m.p: 196–198 °C. ^1^H-NMR *δ*: 2.37 (3H, s), 4.51 (2H, s), 6.88 (1H, d, *J *= 3.9), 7.39 (2H, d, *J *= 8.1), 7.69 (1H, d, *J *= 3.9), 7.97 (2H, d, *J *= 8.1), 11.40 (1H, br s, D_2_O exchangeable). IR cm^−1^: 3421, br (NH), 3043 (CH aromatic), 2916 (CH aliphatic), 2210 (C≡N), 1700 and 1616 (C=Os). Anal. Calcd. for C_18_H_12_BrN_3_O_2_S_2_ (446.34): C, 48.44; H, 2.71; N, 9.41. Found: C, 48.43; H, 2.61; N, 9.73. MS *m/z*: 445 [M]^+^, 447 [M+2]^+^.

*6-Aryl-5-cyano-4-oxo-3,4-dihydropyrimidin-2-yl 2-(4-bromophenyl)-N'-(4-hydroxyphenyl)-2-oxo-ethanehydrazonothioates* **7a**–**c**. An ice cold diazonium salt solution of *p-*aminophenol (prepared from *p-*aminophenol (1.09 g, 0.01 mol), glacial acetic acid (4 mL) and sodium nitrite (0.69 g, 0.01 mol) in water (15 mL)) was added to a chilled solution of the appropriate 6-Aryl-2-(4-bromobenzoylmethylthio)-4-oxo-3,4-dihydropyrimidine-5-carbonitrile **6a**, **6c **or **6e** (0.01 mol) and sodium hydroxide (1.6 g, 0.04 mol) in water (25 mL). The reaction mixture was maintained at −5 °C with continuous stirring for 30 min, and then acidified with glacial acetic acid till pH 5-5.5. The resulting solid was filtered, washed twice with water, dried and crystallized from methanol.

*5-Cyano-4-oxo-6-phenyl-3,4-dihydropyrimidin-2-yl 2-(4-bromophenyl)-N'-(4-hydroxyphenyl)-2-oxo-ethanehydrazonothioate* (**7a**): Yield 65%, m.p: 188–190 °C. ^1^H-NMR *δ*: 6.86 (2H, d, *J *= 8.4), 7.43 (2H, d, *J *= 6.9), 7.54–7.64 (5H, m), 7.66 (4H, d, *J *= 8.1), 10.11 (1H, br s, D_2_O exchangeable), 11.85 (1H, s, D_2_O exchangeable), 13.20 (1H, s, D_2_O exchangeable). IR cm^−1^: 3417–3252 (NH and OH), 3086 (CH aromatic), 2210 (C≡N), 1660 and 1640 (C=Os). Anal. Calcd. for C_25_H_16_BrN_5_O_3_S (546.40): C, 54.95; H, 2.95; N, 12.82. Found: C, 54.93; H, 3.03; N, 12.97. MS *m/z*: 547 [M+2]^+^, 549 [M+4]^+^.

6-(4-Bromophenyl)-5-cyano-4-oxo-3,4-dihydropyrimidin-2-yl 2-(4-bromophenyl)-N'-(4-hydroxyphenyl)-2-oxoethanehydrazonothioate (**7b**): Yield 60%, m.p: 240 °C decompose. ^1^H-NMR δ: 7.41 (2H, d, J = 8.4), 7.63–7.75 (8H, m), 7.92 (2H, d, J = 8.7), 10.00 (1H, br s, D_2_O exchangeable), 10.70 (1H, br s, D_2_O exchangeable), 11.85 (1H, s, D_2_O exchangeable). IR cm^−1^: 3417–3300 (NH and OH), 3089 (CH aromatic), 2214 (C≡N), 1670 (C=O), 1654 (C=O). Anal. Calcd. for C_25_H_15_Br_2_N_5_O_3_S (625.29): C, 48.02; H, 2.42; N, 11.20. Found: C, 48.11; H, 2.45; N, 11.37. MS m/z: 623 [M]^+^, 625 [M+2]^ +^.

*Cyano-4-oxo-6-p-tolyl-3,4-dihydropyrimidin-2-yl 2-(4-bromophenyl)-N'-(4-hydroxyphenyl)-2-oxo-ethanehydrazonothioate* (**7c**): Yield 58%, m.p: 231 °C decompose. ^1^H-NMR *δ*: 2.32 (3H, s), 7.07 (2H, d, *J *= 8.1), 7.34 (2H, d, *J *= 8.4), 7.51 (2H, d, *J *= 8.1), 7.72 (2H, d, *J *= 8.7), 7.85 (2H, d, *J *= 8.4), 7.92 (2H, d, *J *= 8.7), 9.41 (1H, br s), 10.05 (1H, s), 11.80 (1H, s). ^13^C-NMR *δ*: 21.01, 92.45, 115.79, 127.82, 128.42, 128.72, 129.12, 130.23, 131.23, 131.64, 131.74, 131.80, 134.52, 134.81, 141.85, 161.04, 165.03, 166.81, 192.15. IR cm^−1^: 3417–3124 (NH and OH), 3066 (CH aromatic), 2214 (C≡N), 1660 (C=O), 1632 (C=O). Anal. Calcd. for C_26_H_18_BrN_5_O_3_S (560.42): C, 55.72; H, 3.24; N, 12.50. Found: C, 55.78; H, 3.31; N, 12.66. MS *m/z*: 561 [M+2]^+^.

*2-(4-Bromobenzoyl)-5-(4-bromophenyl)-7-oxo-7H-thiazolo[3,2-a]pyrimidine-6-carbonitrile* **9**. A mixture of 6-(4-bromophenyl)-2-(4-bromobenzoylmethylthio)-4-oxo-3,4-dihydropyrimidine-5-carbonitrile **6c** (0.01 mol) and triethyl orthoformate (1.48 g, 1.3 mL, 0.01 mol) in acetic anhydride (10 mL) was heated under reflux with stirring for 8 h. The solvent was concentrated under reduced pressure and the reaction mixture was left overnight. The formed solid was collected, dried and crystallized from aqueous methanol. Yield 40%, m.p: 170–172 °C. ^1^H-NMR *δ*: 7.60 (4H, d, *J *= 8.4), 7.65 (1H, s), 7.82 (4H, d, *J *= 8.4). IR cm^−1^: 3086 (CH aromatic), 2210 (C≡N), 1660 (C=O), 1640 (C=O). Anal. Calcd. for C_20_H_9_Br_2_N_3_O_2_S (515.18): C, 46.63; H, 1.76; N, 8.16. Found: C, 46.71; H, 1.82; N, 8.28. MS *m/z*: 513 [M]^+^, 515 [M+2]^+^.

### 3.2. Biological Evaluation

#### 3.2.1. Determination of the Antimicrobial Activitiess

The antimicrobial activity expressed as MIC, MBC and IC_50_ of tested compounds were determined against four reference bacterial strains; *Staphylococcus aureus *ATCC 6538P, *Bacillus subtilis* ATCC CC33, *Escherichia coli* ATCC 5087, *Pseudomonas aeruginosa *ATCC 9027 as well as against two fungi strains; *Candida albicans* ATCC 60193 *and Aspergillus niger *ATCC 1718109. Amoxicillin, gentamicin and amphotericin B were used as positive control. All assays were conducted in triplicate under strict aseptic conditions. 

##### 3.2.1.1. Determination of the Minimum Inhibitory Concentration (MIC)

The preliminary MICs were firstly determined by the microbroth dilution method [30]. Briefly, 100 µL of double strength DMSO (Sigma-Aldrich, Munich, Germany) were placed in each well of a 96-well microtiter plate. Aliquot of 100 µL of the solutions to be tested were added to the first column. Then 2-fold dilutions were carried out from one well to the next up to final well in each row for each tested compound.

MICs were then determined using agar streaking technique as per Clinical Laboratory Standard Institute guidelines [30]. A total of 15 mL molten (45 °C) Neutrient agar (Sigma-Aldrich) were supplemented with the required concentration then were added into sterilized Petri dishes, allowed to solidify. Then 10 µL of each bacterial or fungal suspension (10^5^ CFU mL^−1^) were streaked onto the surface. Finally all plates were incubated at 37 °C for 24 h for bacterial strains and 25 °C for 48 h fungal strains under aerobic conditions. MIC was determined as the average between the last plate had growth and the first plate with no growth. 

##### 3.2.1.2. Determination of the MBC and IC_50_

MBC and IC_50_ were determined in 96 well microtiter plate where a 100 µL of trypcase soya broth (Oxoid, Lenexa, KS, USA) for bacterial isolates or sabaroud's dextrose broth for fungal strains were placed in each well. A proper amount of the stock solution of the tested compounds was added to reach the desired concentration. All columns were then inoculated with 20 µL of bacterial suspension (10^6^ CFU mL^−1^) and incubated for 5–6 h. An aliquot of 100 µL from each well was transferred into another pre-supplemented with 100 µL f Dey-engly broth medium (Fluka, St. Louis, MO, USA) and allowed to stand for 10–20 min to neutralized any antimicrobial activities. Then these neutralized solutions were subjected to proper dilutions and streaked onto trypcase soya agar or sabaroud's dextrose agar plates to determine the viable count [41]. Controls were done for sterility and growth and subjected to the same regimen of treatment. MBC was determined as the lowest concentration which decreased the number of viable bacteria by 3 log units. IC_50_ was determined as the lowest concentration reduced the viable count by about 50%.

#### 3.2.2. Anticancer Activity [37]

The human tumor cell lines of the cancer screening panel are grown in RPMI 1640 medium containing 5% fetal bovine serum and 2 mM L-glutamine. For a typical screening experiment, cells are inoculated into 96 well microtiter plates in 100 µL at plating densities ranging from 5,000 to 40,000 cells/well depending on the doubling time of individual cell lines. After cell inoculation, the microtiter plates are incubated at 37 °C, 5% CO_2_, 95% air and 100% relative humidity for 24 h prior to addition of experimental drugs. After 24 h, two plates of each cell line are fixed *in situ* with trichloroacetic acid (TCA), to represent a measurement of the cell population for each cell line at the time of drug addition (Tz). Experimental drugs are solubilized in dimethyl sulfoxide at 400-fold the desired final maximum test concentration and stored frozen prior to use. At the time of drug addition, an aliquot of frozen concentrate is thawed and diluted to twice the desired final maximum test concentration with complete medium containing 50 µg/mL gentamicin. Aliquots of 100 µL of the compound dilution is added to the appropriate microtiter wells already containing 100 µL of medium, resulting in the required final compound concentrations. Following compound addition, the plates are incubated for an additional 48 h at 37 °C, 5% CO_2_, 95% air, and 100% relative humidity. For adherent cells, the assay is terminated by the addition of cold TCA. Cells are fixed *in situ* by the gentle addition of 50 µL of cold 50% (w/v) TCA (final concentration, 10% TCA) and incubated for 60 min at 4 °C. The supernatant is discarded, and the plates are washed five times with tap water and air dried. Sulforhodamine B (SRB) solution (100 µL) at 0.4% (w/v) in 1% acetic acid is added to each well, and plates are incubated for 10 minutes at room temperature. After staining, unbound dye is removed by washing five times with 1% acetic acid and the plates are air dried. Bound stain is subsequently solubilized with 10 mM trizma base, and the absorbance is read on an automated plate reader at a wavelength of 515 nm. For suspension cells, the methodology is the same except that the assay is terminated by fixing settled cells at the bottom of the wells by gently adding 50 µL of 80% TCA (final concentration, 16% TCA). Using the seven absorbance measurements [time zero, (Tz), control growth, (C), and test growth in the presence of drug at the five concentration levels (Ti)], the percentage growth is calculated at each of the drug concentrations levels. Percentage growth inhibition is calculated as: [(Ti-Tz)/(C-Tz)] × 100 for concentrations for which Ti >/= Tz or [(Ti-Tz)/Tz] × 100 for concentrations for which Ti<Tz.

## 4. Conclusions

This study reports the synthesis of 6-aryl-5-cyanothiouracil based compounds **4a**–**i**, **6a**–**i**, **7a**–**c** and **9** as potential antimicrobial and antitumor agents. Several newly synthesized derivatives displayed promising antimicrobial activity compared to the reference drugs, amoxicillin, gentamicin and amphotericin B. The activity against gram positive bacteria *S. aureus* and to a lesser extent *B. subtilis* was a characteristic of the majority of active compounds. It can be stated that final compounds **6f** and **6g** with mesomeric electron donating methoxy substituents on the phenyl at the 6 position of the thiouracil scaffoldwere found more active than the other analogs against both Gram positive strains. In addition, the introduction of 4-hydroxyphenylhdrazono moiety in **7b** and **7c** contributed to excellent potency toward the same bacterial strains. On the other hand, *in vitro* cytotoxicity screening of selected compounds **4d**, **4g**–**i** and **6a**–**i** at a single concentration of 10^−5^ M—revealed that most of the compounds had limited cytotoxic activity against renal cancer UO-31 or A498 cell lines. However, compounds **6d** and **6i** exhibited potent growth inhibitory effect toward non-small cell lung cancer HOP-92 and leukemia MOLT-4 cell lines, respectively.

## References

[1] Maga G., Radi M., Gerard M.A., Botta M., Ennifar E. (2010). HIV-1 RT inhibitors with novel mechanism of action: NNRTIs that compete with the nucleotide substrate. Viruses.

[2] Callery P., Gannett P., Williams D.A., Lemke T.L. (2002). Cancer and cancer chemotherapy. Foye’s Principles of Medicinal Chemistry.

[3] Deshmukh M.B., Salunkhe S.M., Patil D.R., Anbhule P.V. (2009). A novel and efficient one step synthesis of 2-amino-5-cyano-6-hydroxy-4-aryl pyrimidines and their anti-bacterial activity. Eur. J. Med. Chem..

[4] Masoud M.S., Ibrahim A.A., Khalil E.A., El-Marghany A. (2007). Spectral properties of some metal complexes derived from uracil-thiouracil and citrazinic acid compounds. SpectrochimActa A Mol. Biomol. Spectrosc..

[5] Fathalla O.A., Awad S.M., Mohamed M.S. (2005). Synthesis of new 2-thiouracil-5-sulfonamide derivatives with antibacterial and antifungal activity. Arch. Pharm. Res..

[6] Odani A., Kozlowski H., Swiatek-Kozlowska J., Brasun J., Operschall B.P., Sigel H. (2007). Extent of metal ion-sulfur binding in complexes of thiouracil nucleosides and nucleotides in aqueous solution. J. Inorg. Biochem..

[7] Prachayasittikul S., Sornsongkhram N., Pingaew R., Techatanachai S., Ruchirawat S., Prachayasittikul V. (2009). Synthesis and novel bioactivities of substituted 6-propylthiouracils. Eur. J. Sci. Res..

[8] Prachayasittikul S., Worachartcheewan A., Nantasenamat C., Chinworrungsee M., Sornsongkhram N., Ruchirawat S., Prachayasittikul V. (2011). Synthesis and structure-activity relationship of 2-thiopyrimidine-4-one analogues as antimicrobial and anticancer agents. Eur. J. Med. Chem..

[9] He Y.P., Long J., Zhang S.S., Li C., Lai C.C., Zhang C.S., Li D.X., Zhang D.H., Wanga H., Cai Q.Q., Zheng Y.T. (2011). Synthesis and biological evaluation of novel dihydro-aryl/alkylsulfanyl-cyclohexylmethyl-oxopyrimidines (S-DACOs) as high active anti-HIV agents. Bioorg. Med. Chem. Lett..

[10] He Y., Chen F., Sun G., Wang Y., Clercq E.D., Balzarini J., Pannecouque C. (2004). Alkyl-2-[(aryl and alkyloxylcarbonylmethyl)thio]-6-(1-naphthylmethyl)pyrimidin-4(3*H*)-ones as an unique HIV reverse transcriptase inhibitors of S-DABO series. Bioorg. Med. Chem. Lett..

[11] Abdel-Mohsen H.T., Ragab F.AF., Ramala M.M., El-Diwani H.I. (2010). Novel benzimidazole-pyrimidine conjugates as potent antitumor agents. Eur. J. Med. Chem..

[12] Galal S.A., Abdelsamie A.S., Tokuda H., Suzuki N., Lida A., El-Hefnawi M.M., Ramadan R.A., Atta M.H.E., El Diwani H.I. (2011). Part I: Synthesis, cancer chemopreventive activity and molecular docking study of novel quinoxaline derivatives. Eur. J. Med. Chem..

[13] Al-Abdullah E.S., Al-Obaid A.-R.M., Al-Deeb O.A., Habib E.E., El-Emam A.A. (2011). Synthesis of novel 6-phenyl-2,4-disubstituted pyrimidine-5-carbonitriles as potential antimicrobial agents. Eur. J. Med. Chem..

[14] Mohamed M.S., Awad S.M., Ahmed N.M. (2012). Anticancer cancer activities of 6-aryl-5-cyano-2-thiouracil derivatives. Pharma Res..

[15] Mohamed M.S., Awad S.M., Ahmed N.M. (2011). Synthesis and antimicrobial evaluation of some 6-aryl-5-cyano-2-thiouracil derivatives. Acta Pharm..

[16] Fathalla O.A., Zeid I.F., Haiba M.E., Soliman A.M. (2009). Synthesis, antibacterial and anticancer evaluation of some pyrimidine derivatives. World J. Chem..

[17] Chen W., Huang Y., Gundala S.R., Yan H., Li M., Tai P.C., Wang B. (2010). The first low μM SecA inhibitors. Bioorg. Med. Chem..

[18] Ding Y., Girardet J.L., Smith K.L., Larson G., Prigaro B., Wu J.Z., Yao N. (2006). Parallel synthesis of 5-cyano-6-aryl-2-thiouracil derivatives as inhibitors for hepatitis C viral NS5B RNA-dependent RNA polymerase. Bioorg. Chem..

[19] Chen H., Tsalkova T., Mei F.C., Hu Y., Cheng X., Zhou J. (2012). 5-Cyano-6-oxo-1,6-dihydro-pyrimidines as potent antagonists targeting exchange proteins directly activated by cAMP. Bioorg. Med. Chem. Lett..

[20] Rose Y., Ciblat S., Reddy R., Belley A., Dietrich L., McKay G., Rafai A., Delorme D. (2006). Novel non-nucleobase inhibitors of *Staphylococcus aureus* DNA polymerase IIIC. Bioorg. Med. Chem. Lett..

[21] Onnis V., Cocco M.T., Fadda R., Congiu C. (2009). Synthesis and evaluation of anticancer activity of 2-arylamino-6-trifluoromethyl-3-(hydrazonocarbonyl)pyridines. Bioorg. Med. Chem..

[22] Edrees M.M., Farghaly T.A., El-Hag F.A.A., Abdalla M.M. (2010). Antimicrobial, antitumor and 5α-reductase inhibitor activities of some hydrazonoyl substituted pyrimidinones. Eur. J. Med. Chem..

[23] Shawali A.S., Elghandour A.H., Sayed A.R. (2001). A Novel One-Pot Synthesis of 3-arylazo-[1,2,4]triazolo[4,3-a]pyrimidin-5(1H)-ones. Synth. Commun..

[24] Bedford G.R., Taylor P.J., Webb G.A. (1995). 15N-NMR studies of guanidines. II-the fused-in guanidine unit of some oxoheterocycles: A combined 15N-NMR, 13C-NMR and IR study. Magn. Res. Chem..

[25] Elguero J., Goya P., Martinez A., Rozas I. (1989). On the tautomerism of 2-phenacyl-4-pyrimidinones and related compounds. Chem. Ber..

[26] Greenhill J.V., Ismail M.J., Bedford G.R., Edwards P.N., Taylor P.J.J. (1985). Conformational and tautomeric studies of acylguanidines. II: Vibrational and carbon-13 nuclear magnetic resonance spectroscopy. Chem. Soc. Perkin Trans..

[27] Reiter J., Bongo L., Dyortsok P. (1987). Synthesis and tautomeric structure of 2-[*N*-aryl-2-oxo-2-arylethanehydrazonoyl]-6-methyl-4(3*H*)-pyrimidinones. Tetrahedron.

[28] Jones R., Rayan A.J., Sternhell S., Wright S.E. (1963). The structures of some 5-pyrazolones and derived 4-arylazo-5-pyrazolones. Tetrahedron.

[29] Shawali A.S., Farghaly T.A. (2004). Synthesis and tautomeric structure of 2-[N-aryl-2-oxo-2-arylethanehydrazonyl]-6-methyl-4(3*H*)-pyrimidinone. Tetrahedron.

[30] Clinical and Laboratory Standards Institute (CLSI) (2009). Performance Standards for Antimicrobial Susceptibility Testing, 19th Informational Supplement.

[31] Nostro A., Roccaro A.S., Bisignano G., Marino A., Cannatelli M.A., Pizzimenti F.C., Cioni P.L., Procopio F., Blanco A.R. (2007). Effects of oregano, Carvacrol and thymol on *Staphylococcus aureus* and *Staphylococcus epidermidis* biofilms. Med. Microbiol..

[32] Prindle R.F., Wright E.S., Block S.S. (1997). Phenolic compounds. Disinfection, Sterilization and Preservation.

[33] Juven B.J., Kanner J., Schved F., Weisslowicz H. (1994). Factors that interact with the antibacterial action of thyme essential oil and its active constituents. J. Appl. Bacteriol..

[34] Monks A., Scudiero D., Skehan P., Shoemaker R., Paull K., Vistica D., Hose C., Langley J., Cronise P., Vaigro-Wolfe A. (1991). Feasibility of a high-flux anticancer drug screen utilizing a diverse panel of human tumor cell lines in culture. J. Natl. Cancer Inst..

[35] Boyd M.R., Paull K.D. (1995). Some practical considerations and applications of the National Cancer Institute in vitro anticancer drug discovery screen. Drug Dev. Res..

[36] Boyd M.R., Teicher B.A. (1997). Cancer Drug Discovery and Development. Anticancer Drug Development Guide: Preclinical Screening, Clinical Trials and Approval.

[37] Skehan P., Storeng R., Scudiero D., Monks A., McMahon J., Vistica D., Warren J., Bokesch H., Kenney S., Boyd M. (1990). New colorimetric cytotoxicity assay for anticancer-drug screening. J. Natl. Cancer Inst..

[38] Parmar J.M., Modha J.J., Parikh A.R. (1999). Synthesis of azetidinones and thiazolidinones from hydrazinopyrimidine as potential antimicrobial agents. Indian J. Chem..

[39] Khodair A.I., Ibrahim E.E., Ashry E.S.H. (1997). Glycosylation of 2-thiouracil derivatives. A synthetic approach to 3-glycosyl-2,4-dioxypyrimidines. Nucleosides Nucleotides.

[40] Abdou I.B., Strekowski L. (2000). A facile synthesis of 6-aryl-5-cyano-1-(β-d-pyranosyl or β-d-furanosyl)-2-thiocytosines. Tetrahedron.

[41] Azéma J., Guidetti B., Korolyov A., Kiss R., Roques C., Constant P., Daffé M., Malet-Martino M. (2011). Synthesis of lipophilic dimeric C-7/C-7-linked ciprofloxacin and C-6/C-6-linked levofloxacin derivatives. Versatile in vitro biological evaluations of monomeric and dimeric fluoroquinolone derivatives as potential antitumor, antibacterial or antimycobacterial agents. Eur. J. Med. Chem..

